# COVID-19 and Emancipatory Gerontology: Perspectives of Political Economy and the American Rescue Plan

**DOI:** 10.1093/geroni/igab046.537

**Published:** 2021-12-17

**Authors:** Carroll Estes, Nicholas DiCarlo, Jarmin Yeh

**Affiliations:** 1 UCSF, University of California San Francisco, California, United States; 2 University of California, San Francisco, institute for Health and Aging, UCSF, California, United States; 3 University of California, San Francisco, San Francisco, California, United States

## Abstract

The present historic moment – a pandemic worsened by far-right extremism – reveals how mounting individual and collective precarity across the lifecourse and in old age resides within societal institutions of colonialism, white supremacy, patriarchy, and capitalism. Contradictions between systems of democracy and capitalism construct an ageist society aligned with neoliberal ideologies attempting to dismantle and privatize Social Security, Medicare, and Medicaid. These issues confront the call for a critical inquiry that matters in the lives of those who daily experience social injustices (Denzin, 2017). This paper presents emancipatory gerontology (Estes & DiCarlo, 2019) as a critical praxis to challenge assumptions, frameworks, and delirium writ large in American society as it relates to how we conceive of age, aging, and generations. We elucidate how the $1.9 trillion 2021 American Rescue Plan represents a paradigm shift that aims to supplant austerity economics with human, public, and community benefit. This knock on the hegemonic commitment to austerity and its mantra is an opportunity to interrogate the effects of, and advance, emancipatory policies and practices. Gerontology is inadequate without a lens for examining how critical analysis and social action might inform one another. To shift from disruption to transformation in the “new normal,” scholars must bring the past and future into the present to engage realistic utopian pedagogies of hope. Emancipatory gerontology offers a theoretical framework and vocabulary for interrogating individual and social consequences of major policy and institutional forces in relation to aging and generations across the lifecourse.

